# Global application of oral disease prevention and health promotion as measured 10 years after the 2007 World Health Assembly statement on oral health

**DOI:** 10.1111/cdoe.12538

**Published:** 2020-05-08

**Authors:** Poul Erik Petersen, Ramon J Baez, Hiroshi Ogawa

**Affiliations:** ^1^ WHO Collaborating Centre for Community Oral Health Programmes and Research University of Copenhagen Copenhagen Denmark; ^2^ University of Texas Health Science Center San Antonio TX USA; ^3^ WHO Collaborating Centre for Translation of Oral Health Sciences Niigata University Niigata Japan

**Keywords:** global policies for oral health, health promotion, oral health systems, prevention, public health

## Abstract

**Objectives:**

The WHO World Health Assembly established in 2007 a Resolution (WHA60.17) on oral health, which called upon countries to ensure that public health actions for disease prevention and health promotion are established. The objective of the present survey undertaken 10 years later (2017‐2018) was to measure the application of such programmes for key population age groups in low‐, middle‐ and high‐income countries.

**Methods:**

Oral health focal points of ministries of health worldwide (n = 101) answered a structured questionnaire on existing national oral health systems and the actual public health activities. The response rate was 58.4%. The questionnaire was used to collect information about structural factors, country workforce, financial models, provision of preventive services and promotion for oral health, school health programmes, administration of fluoride, national oral health targets and oral health surveillance. The countries were classified by national income for analysis of data.

**Results:**

Coverage of population groups by primary oral health care and emergency care varied by national income. The gap between countries in delivery of preventive care was strong since low‐income countries less often reported preventive activities than middle‐income countries and particularly when compared to high‐income countries. School oral health programmes were less frequent in low‐income than other countries. Moreover, population methods of fluoridation and use of fluoridated toothpaste were unusual in low‐income countries. Health education, mass communication and community events were often essential elements in health promotion. In disease prevention, many countries considered the link between oral health and general health conditions and intervention towards shared risk factors of NCDs. The health concern for the consumption of tobacco, unhealthy diet and sugars was particularly emphasized by high‐income countries but less highlighted by low‐income countries. Finally, while national oral health targets for children and surveillance systems were frequently reported by countries, similar systems for adolescents, adults and older people were rare.

**Conclusions:**

The inequities between countries in oral disease prevention and health promotion were substantial. Limited financial resources for preventive care and health promotion; inadequate workforce for oral health, and insufficient coverage in primary health care were observed in low‐resource countries. The results of the survey demonstrate the need for building effective oral health systems oriented towards oral disease prevention and health promotion.

## 1 INTRODUCTION

Many people worldwide suffer from oral disease because of pain and discomfort, loss of function and reduced quality of life.^1^, ^2^, ^3^, ^4^ Oral diseases afflict people of all ages. The World Health Organization (WHO) considers oral conditions, such as dental caries, periodontal disease, tooth loss, oral cancer, HIV/AIDS oral lesions and oro‐dental trauma being significant public health problems.^1^, ^4^, ^5^ As with other noncommunicable chronic diseases (NCDs), poor diet high in sugars, tobacco use and excessive use of alcohol are important risk factors.^3^, ^6^, ^7^ Social determinants are important with poor oral health extraordinary high among deprived population groups^8^; moreover, the disparities in oral health persist across the life course.

Healthcare systems are essential for improving and maintaining the health among population groups. They result from the combined efforts of government agencies, institutions and resources, with the aim of improving health of their people. Properly designed health systems should have a strong component of health promotion and disease prevention, provide for early disease detection and health communication and facilitate appropriate intervention.^9^ A health system requires staff, funds, information, supplies, transport, communication networks, and overall guidance and direction. It also needs to provide services that are responsive and financially fair. Meanwhile, the structure and efficacy of oral health systems vary. Low‐ and middle‐income countries have critical shortages of oral health professionals, and systems are largely symptoms‐oriented. In high‐income countries, oral health professionals are available and advanced systems provide curative and preventive services to people of all ages.^9^, ^10^ Nevertheless, globally, underprivileged people are served inadequately by primary oral health care, and treatment of disease is costly and unfair.^11^

The good news is that the major oral diseases are preventable. Since the 1980s or so, the significance of disease prevention has grown in a number of countries and substantial experience has been gained from implementation of community actions, introduction of preventive activities directed towards population groups and individual preventive care.^12^, ^13^, ^14^ In addition, countries have demonstrated the effect of oral disease prevention through healthy environments. Fluoridation programmes have proven to be effective in preventing dental caries.^15^, ^16^ Health‐promoting schools^17^, ^18^ are successful in encouraging healthy lifestyles and oral health among children and youth. Publicly funded care for older people is shown to improve oral health and quality of life,^19^ and tobacco and alcohol interventions through community actions or dental settings^20^, ^21^ are key actions for reducing oral cancer and periodontal disease. Moreover, healthy diet and reducing the intake of free sugars are important in prevention of dental caries.^22^

### 1.1 WHO policies

Public health intervention against oral diseases of all age groups has gained much concern over the past decades. WHO has given particular emphasis to incorporation of oral health into general health in national NCD prevention programmes and encourages the application of the Common Risk Factors approach.^6^ In 2007, the WHO World Health Assembly called upon countries to ensure that systematic policies for oral health and effective population‐directed oral health programmes are organized. The World Health Assembly established a Resolution (WHA60.17) on *Oral Health: Action plan for promotion and integrated disease prevention*, which stresses the responsibility of countries in developing appropriate public health actions for oral disease prevention and health promotion.^23^ Likewise, WHO pointed out the unique role of the research community^24^ and oral health professionals^25^ in building country capacity for oral health.

### 1.2 Objectives

Ten years after the confirmation of the WHA60.17 resolution on oral health, there is a need to ascertain the current application of policy recommendations on the strengthening of population‐oriented oral disease prevention and health promotion. Thus, the objective of this study was to determine the nature and the extent of appropriate oral health intervention activities for children, youth, adults and older people in low‐, middle‐ and high‐income countries.

## 2 METHODS

The survey took place in 2017‐2018. Chief Dental Officers (CDOs) of Ministries of Health were considered relevant informants for the study, as they are responsible for dental public health and supervision of oral healthcare delivery in their country. They were identified in collaboration with the Global Forum for Chief Dental Officers, the Regional Councils of Chief Dental Officers, WHO and WHO Collaborating Centres in Oral Health, and the World Dental Federation (FDI). Some countries did not have a separate office for oral health, and then related NCD staff undertook the work. Alternatively, public health experts with experience in oral health were selected in a few countries. Of the 194 member states of WHO, it was not possible to identify an officer involved with oral health in 23 small countries. Up to four reminders were used for approaching the 173 potential participants of whom 158 were CDOs.

The global survey covered 101 countries (Figure S1). The countries were classified into national income levels according to the World Bank Atlas Method: ‘low‐income countries’ (21 countries), ‘middle‐income countries’ (39 countries) and ‘high‐income countries’ (41 countries). The response rate for countries as a whole was 58.4%; the participation rate of low‐income countries was 51.2%; 50.3% for middle‐income countries and 74.6% for high‐income countries.

A structured questionnaire was designed for gathering information on existing national oral health systems and actual preventive activities. The following variables were included: structural factors (population data, national income in terms of Gross National Product (GNP in US Dollars), percentage of GNP spent on health care and oral health care), country workforce, financial models, organization of work for oral health, school health programmes, community approaches to oral health, fluoride administration, and national oral health targets and surveillance. The questionnaire was pretested by a group of public health administrators from low‐, middle‐ and high‐income countries.

Over the years, countries have been encouraged by national and international health authorities like WHO to accumulate quality oral health data regularly in order to undertake oral health surveillance, to facilitate health policy development and to advance building of appropriate oral health systems.^23^ In this survey, the participants were asked to report on availability of recent national information about oral health status of the key population groups; these data were used for portraying the level of country oral health. Responders were invited also to provide information on available epidemiologic reports/references relevant to establishment of oral health figures for their country. As part of the data analysis, the responses were checked against data obtainable from the WHO Global Oral Health Data Bank.^26^ The latter also permitted inclusion of epidemiological data for some countries not taking part in the questionnaire survey. In conclusion, countries showing oral health figures from the year 2000 and onwards were considered relevant to the analysis of epidemiological data and to the creation of global oral health maps.

Analysis of data collected by the survey included univariate, bivariate and multivariate frequency distributions, and statistical tests were carried out by chi‐square test, Student's *t* test and ANOVA. The levels of statistical significance of results are presented in the tables.

## 3 RESULTS

### 3.1 Resources for oral health

Table S1 presents the average level of GNP used up on health and oral health per year. In total, countries spent nearly 8% of GNP on health while about 1% of GNP was allocated for oral health. For both general health and oral health, the GNP allotment was relatively lower in low‐income countries than in high‐income countries.

The information about oral health workforce available in countries is shown in Table S1. In low‐income countries, the mean number of registered dentists was low, whereas the figure was substantially higher in middle‐ and high‐income countries. Half the dentists in low‐income countries worked in public health service against one fifth of dentists in high‐income countries. Seven in ten dentists in high‐income countries worked in private dental practice. The population per dentist measure ranged from 152 721:1 in low‐income countries to 1708:1 in high‐income countries. In addition, the availability of dental auxiliary personnel was prominent for high‐income countries, where the average number of chairside assistants per dentist was 1.5:1; the figure was low in low‐income countries. Oral hygienists were primarily found in high‐income countries.

### 3.2 Financial models

Table S2 presents the percentage of countries where population groups receive oral health care paid by government, partial support from government or care provided with no contribution by government. The findings are given for each of the key population groups. For example, 9.5% of low‐income countries reported that preschool children received governmental care, 4.8% of countries stated that they offered children some support through government sources while 85.7% indicated no such support. By contrast, 31.7% of high‐income countries reported that preschool children were covered by governmental care whereas 51.2% of countries declared that young children did not get such support. For high‐income countries, the support to oral health from government was fairly low for adults and older people.

The involvement of private health insurance in oral health care for population groups is illustrated in Table S3. For schoolchildren, for example, 38.1% of low‐income countries reported that children were supported from private insurance while 61.9% of countries reported that schoolchildren had no such support. In parallel, the insurance cover for schoolchildren was specified by 26.8% of high‐income countries. One third of countries reported that private insurance was involved in oral health care of adults and older people.

Table S4 presents data on direct payment for oral health care. For child populations and adolescents, about four in ten countries had direct payment systems. In approximately half the countries, adult people had to pay for themselves directly and that proportion tended to be slightly higher in high‐income than in low‐income countries.

### 3.3 Provision of preventive care

Table S5 presents data on provision of primary oral health care. About half of the low‐income countries reported that preschool children were covered by primary oral health care against nearly three‐quarters of high‐income countries. The response pattern was similar for other population groups; it is worth noting that the variation in primary oral healthcare coverage by national income was substantial for older people. Nearly all high‐income countries offered access to emergency care for children and adolescents, whereas that was lower in low‐income countries.

Table S6 indicates the proportion of countries informing that they delivered specific programme activities in prevention of oral disease. Dental examinations for schoolchildren and adolescents were less frequent in low‐income countries than in high‐income countries. In all, about half of countries reported use of topical fluoride application for preschool children; such application was used in schoolchildren by 7 out of 10 countries and 4 out of 10 countries for adolescents. Topical fluoridation programmes were less common in low‐income countries. Oral hygiene and health education programmes for children and adolescents were common but less so for children in low‐ and middle‐income countries.

Table S7 highlights the existence of preventive activities for adults and older people. About 40% of countries informed that adults and older people were offered dental examinations; around one fifth of all countries stated that they had programmes offering topical fluoride application, while half the countries indicated that they provided health education for adults and older people. Preventive care for adults and older people was less common in low‐income countries.

### 3.4 School‐based oral disease prevention and health promotion

Three in four countries had school programmes for oral health though they were less frequent in low‐income countries (Table S8). One third of countries offered fluoride mouth rinsing in schools for children aged 5‐7 and 12 years. Moreover, pit and fissure sealing were used for dental caries prevention, particularly among children aged 5‐7 in 70.4% of the countries and in 59.2% of countries for children of age 12. Some countries reported that children were offered treatment services in schools; notably, this was indicated for children of ages 5‐7 (60.6%) and 12 years (64.8%) but less frequent in low‐income countries.

Table S9 illustrates the proportion of countries providing oral health education to children through schools. About two thirds of countries had health education for children aged 5‐7 years and 12 years, which emphasized proper nutrition, healthy diet, and issues related to consumption of sugars. Approximately half the countries reported having tobacco‐focused activities for 12‐ and 15‐year‐olds, somewhat more frequent in high‐income countries. Approximately 4 in 10 countries had preventive programmes in relation to alcohol.

### 3.5 Community‐oriented programmes for oral health

Table S10 presents the proportion of countries engaged in different community activities for oral health. Some 40% reported that mass communication often or very often was used for promoting oral health, and about half the countries reported that community campaigns or events for oral health were organized regularly. Maternity and child health facilities were chosen as a platform for oral health communication by 44.9%; however, this activity was less common in low‐income countries. One third of countries stated that they often communicate with vulnerable families for better oral health. Nearly 20% reported having programmes for oral health of older people, but they were less common in low‐income countries (4.8%).

Table S11 shows the proportion of countries having initiated a broad approach to disease prevention and health promotion. Half the countries indicated that their national NCD programme was concerned about oral health in the control of diabetes and around 40% mentioned the importance of cardiovascular diseases. Oral health was also considered in tobacco prevention programmes by two thirds of countries though this concern was less common in low‐income countries. In all, just over half of countries had focus on the impact of unhealthy diet and sugars and a similar proportion of countries had a national policy or official recommendations for reducing the intake of free sugars. Meanwhile, a national policy for reducing the intake of free sugars was less common in low‐income countries.

### 3.6 Fluoride for oral health

The responses to questions on use of fluoride for prevention of dental caries are summarized in Table S12, showing the mean percentage of national populations benefitting from fluoride programmes. Accordingly, low‐income countries reported that no population groups were exposed to adequate fluoride in drinking water (natural or artificial). On average, 7.6% of people in middle‐income countries were reported to have benefit from fluoride in drinking water, while the figure counted 20.9% for high‐income countries. On average, 30.8% of people in low‐income countries had benefit from fluoride in toothpaste, 52.1% of people in middle‐income countries, against 81.5% of people in high‐income countries. Fluoridated salt or milk was relatively seldom in all countries.

One fourth of respondents informed that the political interest in water fluoridation was good or very good; however, the interest in salt fluoridation or milk fluoridation was somewhat lower. The respondents were asked whether essential conditions for population methods of fluoridation (water, salt or milk) were encountered in their country. Almost half the respondents referred to a high burden of dental caries, about one fourth reported that technical requirements and facilities were available, while nearly one third of respondents declared that the necessary expertise existed in their country. Finally, the respondents were asked whether their country had any plans of introducing population methods fluoridation programmes. Plans about water fluoridation were confirmed by 16.3% of countries, 12.4% for salt fluoridation, and 6.2% of countries mentioned milk fluoridation. Salt fluoridation was considered particularly by low‐income countries.

Table S13 presents the percentages of countries with specific recommendations on concentration of fluoride in toothpaste for children and adults. Toothpaste with a low level of fluoride (400‐600 ppm F) was suggested for use in preschool children by approximately 40% of countries. In high‐income countries, toothpaste with fluoride at the level of 1000‐1500 ppm F was recommended for use in preschool children by nearly 50%. The majority of high‐income countries recommended this type of fluoride‐containing toothpaste for use in schoolchildren and adults, while such recommendation was significantly lower for middle‐ and low‐income countries. Several low‐income countries (45%) reported that quality recommendations for toothpaste did not exist.

### 3.7 Targets and information systems for oral health

Half the countries reported having oral health targets for children aged 5‐6 and 12 years; meanwhile, targets for oral health of 12‐year‐olds were relatively less frequent among low‐income countries (Table S14). Targets for oral health of adolescents and adult age groups were stated by lower percentages; one fourth of the countries had oral health targets for adults aged 35‐44 whereas one fifth of countries had targets on oral health for older people.

Elements of oral health targets as formulated by countries were measured by an open‐ended question. The major components of oral health targets for children were dental caries prevalence (primary and permanent dentitions); reduction of disease prevalence rates; reducing the unmet need for dental care (d/D index components); prevalence of dental erosion; risk factors such as sugars; dental care; treatment services; and school oral health programmes. Additionally, targets for oral health of adults focused on reducing the burden periodontal disease; oral cancer screening; tobacco; quality of care; oral health of pregnant women; oral health awareness of the general population; and improvement of healthy lifestyles. Targets for oral health of older people also included actions for a functional dentition of adults; reducing the prevalence of tooth loss; avoiding root caries, and controlling the need for prosthetic dental care. Reference was often made to WHO indicators; WHO policies on oral health and NCD prevention; health promotion programmes; social determinants of oral health; and primary oral health care.

Respondents were enquired about the existence of a national information system for oral health surveillance. Systems for children (58.2%) and youth (50.0%) were fairly often reported, while systems for adults (38.8%) and older people (35.7%) were less frequent. Information systems for children and youth were commonly indicated by high‐income countries.

### 3.8 Oral health status

Respondents were asked about the recent country figures on oral health status of the WHO key population groups: children aged 5 or 6 years, 12‐year‐olds, 18‐year‐olds, adults aged 35‐44, and people aged 65‐74/65+. In addition to the participating 101 countries, dental caries data on children aged 12 were supplementary identified from 17 countries as recorded in the WHO Oral Health Data Bank.^26^

As shown in Table S15, in low‐income countries, 36.3% of 5‐ or 6‐year‐olds were caries‐free while the figure was relatively higher (50.2%) for high‐income countries. The level of dental caries experience (DMFT) among 12‐year‐old children was particularly high in middle‐income countries. Dental caries experience showed relatively high levels among young adults and older people in high‐income countries.

Information about dentate status and periodontal health conditions was seldom recorded in low‐income countries (Table S16). Worth noting, figures on older people with 20 teeth or more were lower in middle‐income than in high‐income countries. On average, half the persons had teeth with gingival bleeding among people aged 15‐19 and 35‐44 years, and an estimated 9.1% of 35‐44‐year‐olds and 17.4% of older people had deep periodontal pockets. Relatively small differences in adult periodontal health status were reported by national income.

### 3.9 Global oral health maps

Six global maps were prepared to provide a geographical overview of oral health status of the key population age groups. The intention was to portray the oral health situation of countries by prevalence or severity level of disease. Figures S2‐S7 indicate the epidemiological measures and categories.

Country data were readily available for dental caries among children and adults aged 35‐44 years. In Eastern Europe and parts of Africa and Latin America, most countries had less than 50% of 5‐6‐year‐olds without dental caries. For children aged 12, DMFT values generally were either low or very low. Data on dental caries of older people were scarce and only a limited number of countries had data on dentate status.

## 4 DISCUSSION

Opposite to the existing WHO Global Oral Health Data Bank,^26^ WHO has not established a databank on delivery of oral health services in countries. The present questionnaire survey involved a large number of countries around the world and they provided for thorough analyses of existing oral health programmes in low‐, middle‐, and high‐income countries. It is worth noting that a cross‐sectional design was applied for the study, and therefore, it was not possible to assess the number of countries having introduced or modified existing programmes in response to the WHA.60.17.

In all, an acceptable response rate for the survey was obtained. The response to the survey was approximately the same as obtained in a recent WHO global survey of oral health through schools.^18^ Of all responses given by national CDOs, NCD staff, or public health administrators, 39.1% were received from high‐income countries, 49.1% were received from middle‐income countries, while 11.8% came from low‐income countries.

Notably, the response rate was high in high‐income countries while the rates were somewhat lower in low‐ and middle‐income countries. Some selection bias might be expected. Participation was very good in countries of Europe and North America. The response of Asian countries was acceptable whereas the response rate was particularly lower in countries of Africa and Latin America. This may be due to several factors; primarily the absence of a country focal point for oral health, the capacity in oral health administration may be limited, or the public health interest of potential informants may be low. The survey indicated that oral health systems and preventive practices were less structured in countries of these geographical areas; consequently, the findings may display a somewhat positive picture as to the existence of programmes in low‐ and middle‐income countries.

The questionnaire was formulated in English. Obviously, responders of countries have different languages. The pretest, which took place in a number of low‐, middle‐, and high‐income countries, considered the potential language barrier in understanding the specialized expressions applied. Although language might be a source of information bias, the questionnaire used basic expressions applied in preventive dentistry, which are part of the standard professional terminology across the world.

Due to practical and economical concerns, a computer‐assisted questionnaire was used to collect data for the study. The structured questionnaire also included semi‐structured or open‐ended questions. However, compared to personal interviews, self‐completion questionnaire implies some principal disadvantages. There is no one present to help respondents if they have technical difficulties answering questions; there is no opportunity to probe respondents to elaborate an answer; you can never be sure whether the right person has answered the questionnaire; long‐questionnaire is rarely feasible; and there is a greater risk of incomplete answers or missing data. The Chief Dental Officers (CDOs) invited for the study are experienced in questions about oral health administration and supervision of oral healthcare programmes as implemented at national level. Therefore, it is anticipated that the information collected on oral disease prevention and health promotion is fairly accurate and reliable. In addition, CDOs are acquainted with population oral health statistics in their country.

### 4.1 Resources for oral health

Information about economic input and health workforce is essential for understanding the output of oral health systems.^27^ This survey highlighted the country investment for health in terms of the proportion of GNP spent on health. The country variation in GNP spent on health is consistent with the figures reported recently by WHO.^28^ In all, 1.3% of the annual GNP was allocated for oral health; however, high‐income countries showed the highest figure.

The shortage of dentists in low‐ and middle‐income countries is critical to serving the population in oral health care. Particularly, the deficiency in the number of dentists in low‐resource countries is a major barrier to capacity building in public health and the organization of population‐oriented programmes for oral disease prevention and health promotion.^10^, ^29^ As reported, the number of dentists is higher in high‐income countries, and chairside assistants and oral hygienists may facilitate the delivery of preventive services.

### 4.2 Financial models

Financial systems affect the establishment of preventive and restorative services and they may also distress access to oral health care.^9^, ^30^, ^31^, ^32^ In this survey, only one fifth of all countries had government care for children and adolescents. Notably, government involvement was particularly weak in low‐income countries. For all countries, three in ten reported that people received some support from private health insurance, and direct payment for oral health care (in full or partial) was common. Thus, the delivery of oral health care is left largely to private sources entailing significant barriers to universal coverage and to the establishment of prevention and health promotion programmes. Third‐party payment and capitation systems occur primarily in high‐income countries and they generally have focus on individual care. The *Cochrane Collaborati*on^33^ confirmed recently the strong incentives of third‐party payment towards delivery of preventive dentistry. Capitation systems may stimulate preventive advice and application of fissure sealants.^32^

### 4.3 Primary oral health care

Major inequities between countries were shown in provision of primary oral health care and accessibility to emergency care. The inadequate number of oral health professionals in low‐ and middle‐income countries implicates incomplete access to primary oral health care.^10^ In addition, absence of proper facilities and high costs of oral health care may result in low quality of care and population groups being underserved. The WHO policy on Universal Health Coverage^34^ emphasizes that countries should ensure that all individuals and communities receive the quality health care they need without suffering financial hardship. It includes the full range of essential health services from health promotion to prevention and treatment. The realization of this policy as to oral health remains an extraordinary challenge to low‐resource communities or countries.

### 4.4 Specific oral disease prevention

The evidence is strong on various actions of population‐directed oral disease prevention and health promotion.^35^, ^36^, ^37^ Reports confirm the empirical evidence of dental examinations^38^; topical application fluoride^16^, ^39^; oral hygiene instruction^40^, ^41^; pit and fissure sealing^42^; community participation^43^; personal health education with focus on a healthy diet^44^; reducing the intake of sugars and promoting good nutrition^22^; and avoiding the use of tobacco.^13^, ^44^ Moreover, the effectiveness of preventive dentistry is demonstrated in reducing the amount of dental treatment and related expenditures on dental care.^45^ The intention of this survey was not to judge at what level people are actually benefitting from preventive care. The concept was to highlight the actual employment of evidence‐based preventive services and health promotion offered to key population groups under existing national health programmes. The results are striking as major inequities between countries were shown in the delivery of preventive care for all population groups. This gap may be ascribed to the several health systems factors. These include absence of policies for oral health; inadequate workforce for implementation of preventive care; insufficient financial support to prevention in low‐ and middle‐income countries; and low encouragement of dentists to application of prevention alongside the incentives of restorative care or even radical care.

### 4.5 School oral health programmes

School health education is the most important component of health promotion aiming at improving health and creation of healthy lifestyles. The majority of high‐ and middle‐income countries reported having school oral health programmes, while they were less frequent in low‐income countries. Fluoride mouth rinsing for schoolchildren was indicated by one third of countries while the differences between countries were minor. Country inequities were, however, found for pit and fissure sealants of permanent teeth to schoolchildren as such service was less used in low‐income countries. Many high‐ and middle‐income countries offered dental treatment in schools whereas this was less frequent in low‐income countries, probably because of lack of resources.^18^ Importantly, education programmes covered health communication in good nutrition, healthy diet, and information about the negative impact of consumption of sugars, though differences between countries were found for tobacco prevention.

### 4.6 Community approaches

An increase in the awareness of oral health is a primary effect from use of mass media while improvement of health behaviour is less convincing.^44^ All countries often or very often made use of mass communication but it is noteworthy that this type of communication was more frequent in low‐income countries. Community campaigns and events have proven useful in engaging people in oral health^46^ and the present study interestingly showed that such activities were organized on a regular basis by all countries. Maternity centres are key community settings for tackling health problems, particularly of children, young mothers and pregnant women.^47^ Such centres are mostly useful in provision of aid, control of acute symptoms, health communication and advice in health care to mothers. Despite the experience,^48^ maternity facilities were rarely used as a platform for oral health by the participating low‐income countries.

High risk of disease is found for certain population groups.^8^ Poor oral health is prevalent among vulnerable families^48^and older people,^49^, ^50^ but surprisingly few countries reported having community programmes for disadvantaged people. The working place represents a unique setting for preventive programmes and experiences have been gained in avoiding occupational oral diseases.^51^ Only a few countries reported having preventive activities initiated from the working place.

### 4.7 NCD prevention and oral health

The so‐called ‘Common Risk Factors approach’^6^ is widely accepted in dental public health. This approach links prevention of oral diseases with the fight against chronic diseases, primarily through intervention towards modifiable risk factors such as intake of sugars, use of tobacco and excessive alcohol drinking.^52^, ^53^ The strategy focuses on the underlying social determinants of health and encourages oral health workers to work in partnership across sectors and disciplines. Moreover, the model embraces the interaction between oral health and general health, such as the reciprocal direct links between periodontal health and diabetes mellitus and the association between periodontal disease and cardiovascular diseases.^54^, ^55^

Interestingly, a substantial number of countries replied that they had national NCD programmes, which covered prevention of oral diseases as well. Furthermore, many countries considered the link between compromised nutrition, obesity and dental caries in disease prevention.^56^ The use of tobacco and excessive consumption of alcohol are primary causal factors of oral cancer and periodontal disease.^57^ Two thirds answered that they considered oral health in tobacco prevention while alcohol prevention had a lower score.

Several high‐income countries addressed diet and sugars in prevention of NCDs and official recommendation existed often for reducing the intake of sugars. Such recommendation is in agreement with the WHO sugar policy,^22^ which also calls for actions to be undertaken by oral health professionals.^58^ Some low‐ and middle‐income countries indicated having policies against consumption of sugars^59^; however, the concern for diet was less common in these countries. Thus, it is desirable that countries consider the WHO sugar policy for better health.^22^

### 4.8 Fluoride and oral health

Administration of fluoride through the environment is a unique opportunity for prevention of dental caries. The fluoridation of drinking water is renowned as an effective, safe and economically beneficial public health measure for disease prevention.^16^, ^60^ Meanwhile, lack of piped drinking water impedes installation of water fluoridation in many communities globally. Adding fluoride to salt may be an alternative to water fluoridation and milk fluoridation is relevant to disease prevention among children, particularly when linked to an existing school health programme. Importantly, fluoride‐containing toothpaste is the most widely used method of applying fluoride,^16^ and it is recommended for disease prevention at the fluoride level of 1000‐1500 ppm whereas high concentration of fluoride (>1500 ppm) may have relevance to people suffering a high risk of dental caries.

About one fifth of people in high‐income countries were exposed to fluoride in drinking water (full or part of population), whereas one tenth of people in middle‐income countries reported the benefit from fluoride in salt; fluoridated milk was seldom. In low‐income countries, it is remarkable that less than one third of people on average benefitted from toothpaste containing fluoride. This was the case for some 50% of people in middle‐income countries but more than 80% in high‐income countries. Toothpaste of low concentration of fluoride (400‐600 ppm F) was used for young children by several countries but the preventive effect of this type is uncertain.^16^ Thus, promoting oral health of children, adolescents and adult people in low‐ and middle‐income countries through the use of fluoridated toothpaste of optimal quality (>1000 ppm F) is a matter of urgency.

The majority of high‐income countries had official recommendations on effective toothpaste containing fluoride; in comparison, nearly half the low‐income countries and one third of middle‐income countries had no such recommendations. It is an important experience that recommendation of fluoridated toothpaste is essential to development of national policies on effective use of fluoride.^16^

Significant proportions of respondents informed that population methods of fluoridation existed because of a high burden of dental caries, that technical requirements and facilities were available in the country, and that the necessary country expertise for establishment of population methods of fluoridation was available. These specific conditions were less frequent in low‐ income countries; meanwhile, one fifth of the countries mentioned that they had plans of introducing water or salt fluoridation programmes. In all, the political interest in water fluoridation was higher than for salt or milk fluoridation.

### 4.9 Oral health targets and information systems for surveillance of oral health

WHO, the World Dental Federation (FDI) and the International Association for Dental Research (IADR) published in 2003 a series of global oral health indicators for the measurement of national and global targets by the year 2020.^61^ The oral health indicators included measures on dental caries, periodontal disease, tooth loss, the creation of human resources for oral health care and percentage of the population with access to adequate primary oral health care. Health authorities across the world have accepted these indicators and they are now used successfully by countries for formulation of targets for oral health and establishment of surveillance systems.

The respondents of this survey were asked if national oral health targets were formulated for key population groups. While nearly half the countries had targets in relation to children aged 5‐6 and 12 years, only 20%‐25% of countries expressed having targets for adolescents and adult age groups. Differences between countries in answers on oral health targets were minor. For countries with expressed targets, components often focused on standards of oral health status to be achieved in the future, oral health systems adjustment and building of adequate primary oral health care, quality of life and health behaviour modification.

The oral health indicators suggested by WHO^62^ have permitted national surveillance and international comparison over time. A significant proportion of high‐ and middle‐income countries reported that they had a national information system for surveillance of oral health in children though this was the case for a smaller number of the low‐income countries. In all, national information systems for oral health in adults and older people were less common.

Even with WHO recommendation, statistics on the occurrence of oral disease were limited in low‐income countries; this probably reflects a shortage of public health researchers and a weaker attention by health authorities. The information was considerable in middle‐ and high‐income countries. The survey pointed to a relatively high proportion of caries‐free 5‐ to 6‐year‐olds in high‐income countries while dental caries experience among children aged 12 was prominent in middle‐income countries. This is in agreement with previous reports on global oral health of children.^4^ Remarkably, the dental caries figures of 35‐ to 44‐year‐olds and older people were manifest for high‐income countries. Likewise, the information on periodontal health status (CPI) was scarce in low‐income countries. In middle‐ and high‐income countries, among adolescents and 35‐ to 44‐year‐olds, approximately half the people had teeth affected by gingival bleeding. Shallow pocketing was reported for one fourth of people aged 35‐44, whereas deep periodontal pockets were specified for nearly one fifth of people aged 65‐74.

### 4.10 External validity of epidemiological findings

The WHO Global Oral Health Data Bank^26^ stores worldwide information about oral health status. The bank includes adequate data from a number of countries allowing for assessment of oral health status over time. In particular, data on dental caries experience (DMFT) of 12‐year‐olds have been available since 1980; the information demonstrates that the burden of dental caries declined from an average of 2.42 DMFT in 1980 to the level of 1.86 DMFT in 2015. Importantly, the present survey of countries has obtained a similar average figure of 1.87 DMFT and the consistency of findings therefore may indicate a satisfactory external validity of the epidemiological data on dental caries.

### 4.11 Global oral health maps 2017‐2018

The first WHO global maps on oral health focused on dental caries (DMFT) in children aged 12 and adults aged 35‐44; they were primarily based on data provided by CDOs or national public health researchers during the late 1990s. Oral health data available were then used to classify countries according to the level of dental caries and they were published by WHO in the World Oral Health Report 2003.^63^ An update of the map on dental caries of 12‐year‐olds took place in 2014.^26^

The global maps prepared for the present report were based on comprehensive country data reported for the year 2000 and onwards. The respondents were requested information about the national representative values of oral health conditions and they were also asked to provide information about any recent national reports. Such survey information permitted an assessment of potential critical issues of parameters, such as source of statistics, geographical area, socio‐economic status, number of people examined, diagnostic criteria, and number of examiners and training.^64^

The classification criteria of the dental caries levels used for the age groups were stable over time. Moreover, the number of maps was extended to cover all the WHO indicators and population groups, that is including the specific oral health conditions of people aged 5 or 6 years, 12 years, 35‐44 years and 65‐74/65+ years.

The actual figures indicate that a low level of caries‐free children aged 5‐6 was frequent in certain countries of Eastern Europe, the Middle East, Asia, Africa, and Latin America. North America and Western Europe had moderately high levels of caries‐free children while northern Europe showed the highest scores. When compared to the equivalent maps of the year 2002^63^ and 2014,^26^ the actual map of 12‐year‐olds points to an increasing number of countries with very low or low levels of DMFT, which may reflect an introduction of preventive programmes over the past decades.

Traditionally, the severity of dental caries among young adults and older people has been high in many industrialized countries.^1^, ^2^, ^3^, ^4^ However, this survey indicated a growing number of countries with moderate levels of dental caries. Information about older people with at least 20 natural teeth, respectively, older people without any natural teeth might have been useful in assessing measures in relation to quality of life^62^; meanwhile, it is unfortunate that country data on dentate status were rather scarce.

## 5 CONCLUSION

The intention of the present global survey was to determine country application of oral disease prevention and health promotion 10 years after the approval of the 2007 WHO World Health Assembly resolution on oral health.^23^

Inequities between countries in coverage by primary oral health care and emergency care is substantial. The study indicated that it remains a challenge to especially low‐ and middle‐income countries to realize the WHO policy on Universal Health Coverage as regards oral health and as recommended by the 2019 United Nations Political Declaration on Universal Health Care.^65^

A considerable number of countries have established programmes for oral disease prevention and health promotion. Meanwhile, the gap between countries in delivery of preventive services and health promotion is strong, which may relate to health systems factors. In low‐and middle‐income countries, these factors include insufficient financial support to prevention and health promotion; inadequate workforce for oral health; and absence of policies for oral health.

The establishment of school‐based oral health promotion varies significantly between countries. Preventive services and health promotion in schools have the potential of breaking existing health inequities in children and adolescents and oral health through schools should be ensured by countries.

The use of maternity and child health facilities is occasional in low‐income countries. Such centres are important for health communication, disease prevention and control and should be reinforced.

According to the survey, several countries have accepted an integrative approach for oral disease prevention and health promotion. Effective disease prevention links oral health with general health and intervention against NCD risk factors. Intervention against tobacco use and consumption of sugars needs strengthening in low‐income countries. National policies for reduction of the intake of sugars should be encouraged for countries without such authorized recommendations.

Most people of high‐income countries benefit from use of toothpaste containing fluoride; however, enhanced use of such toothpaste is desirable in low‐ and middle‐income countries. Fluoridation methods should be considered by countries where people are not exposed adequately to the preventive effect of fluoride. Such initiative may help breaking the inequities in dental caries. Optimal concentration of fluoride in toothpaste is warranted and should be recommended by national health authorities.

Formulation of national targets for oral health and establishment of information systems for surveillance of oral health of children, adolescents, adults and older people should be encouraged.

## AUTHOR CONTRIBUTIONS

All authors have made substantial contributions to the design of the study, data collection, interpretation of data and final approval of the version to be published.

## Supporting information

Figures S1‐S7

Table S1‐S16
