# Supplementary material for: Global application of oral disease prevention and health promotion as measured 10 years after the 2007 World Health Assembly statement on oral health
Source: Community Dent Oral Epidemiol. 2020 May 8;48(4):338–48. doi: 10.1111/cdoe.12538 (PMC7496398; doi:10.1111/cdoe.12538)
Supplement: Supplementary file 1 — Figures S1‐S7 [file CDOE-48-338-s001.pdf]

Global application of

Oral Disease Prevention and Health Promotion

as measured ten years after the 2007 WHO World Health Assembly statement on oral health

Online supplementary information

Figures S1-S7

Correspondence:

Professor Poul Erik Petersen, DDS, Dr.Odont.Sci. BA, MSc (Sociology)

WHO Collaborating Centre for Community Oral Health Programmes and Research

University of Copenhagen

DK-1014 Copenhagen K, Denmark

poep@sund.ku.dk

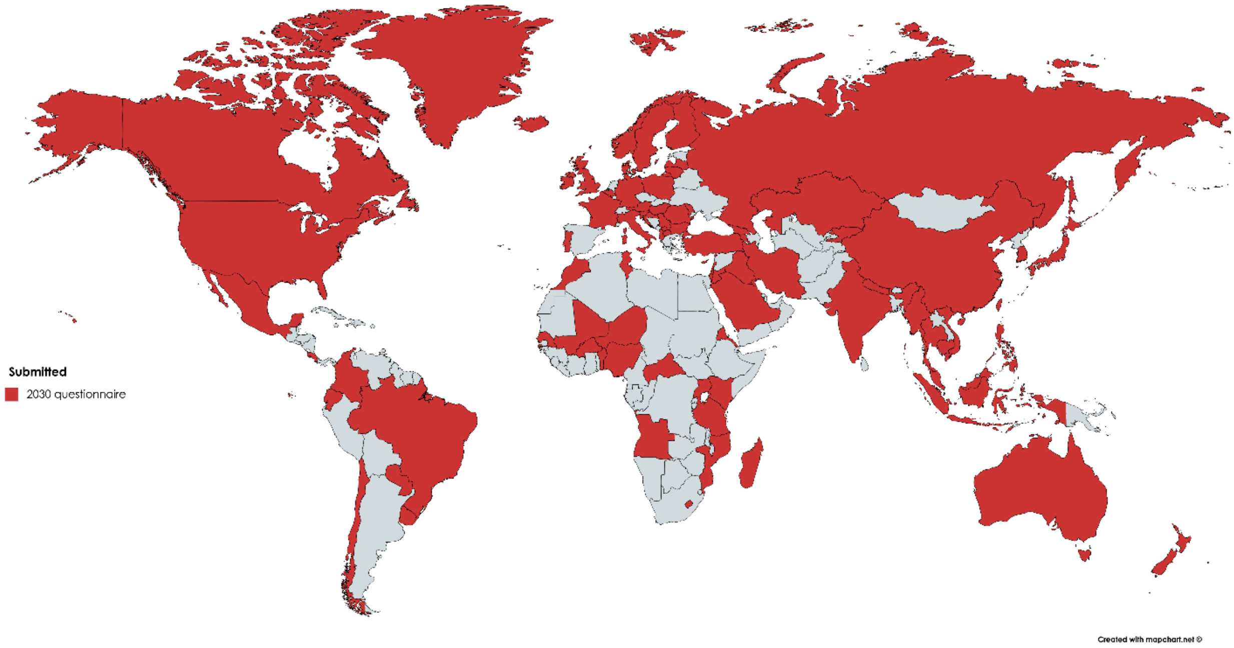

Fig. S1 Countries participating in the global survey of oral disease prevention and health promotion.

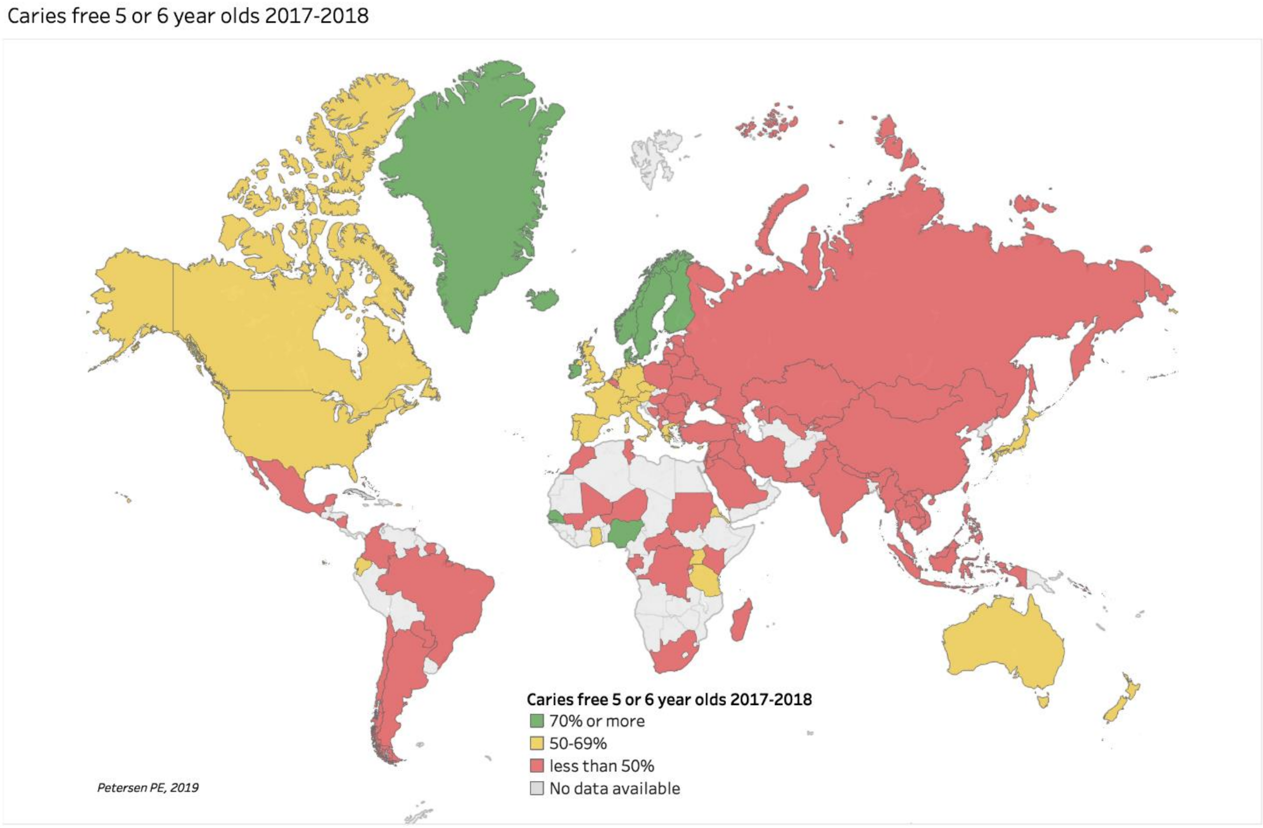

Fig. S2 Percentage of the child population aged 5 or 6 years being free of dental caries in countries across the world.

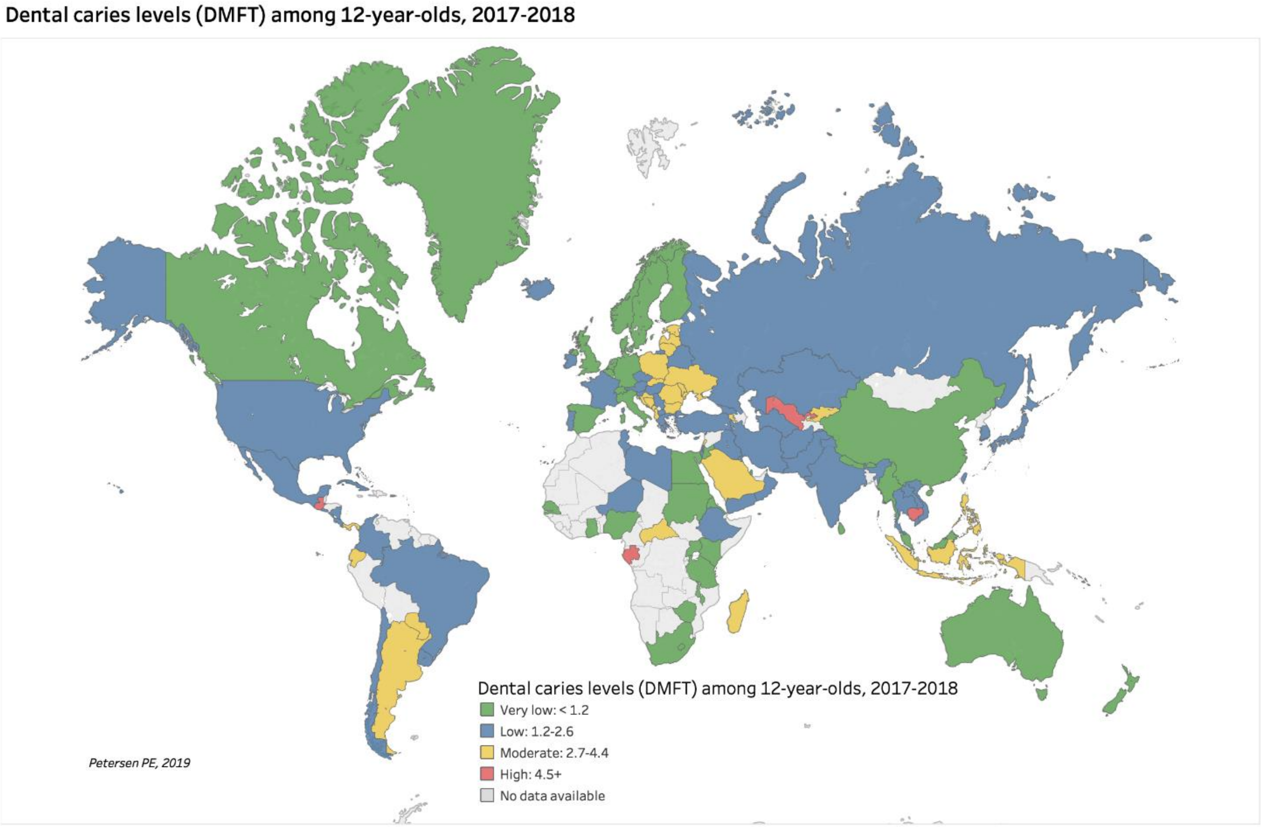

Fig. S3 Mean number of permanent teeth affected by dental caries (DMFT) in children aged 12 in countries across the world.

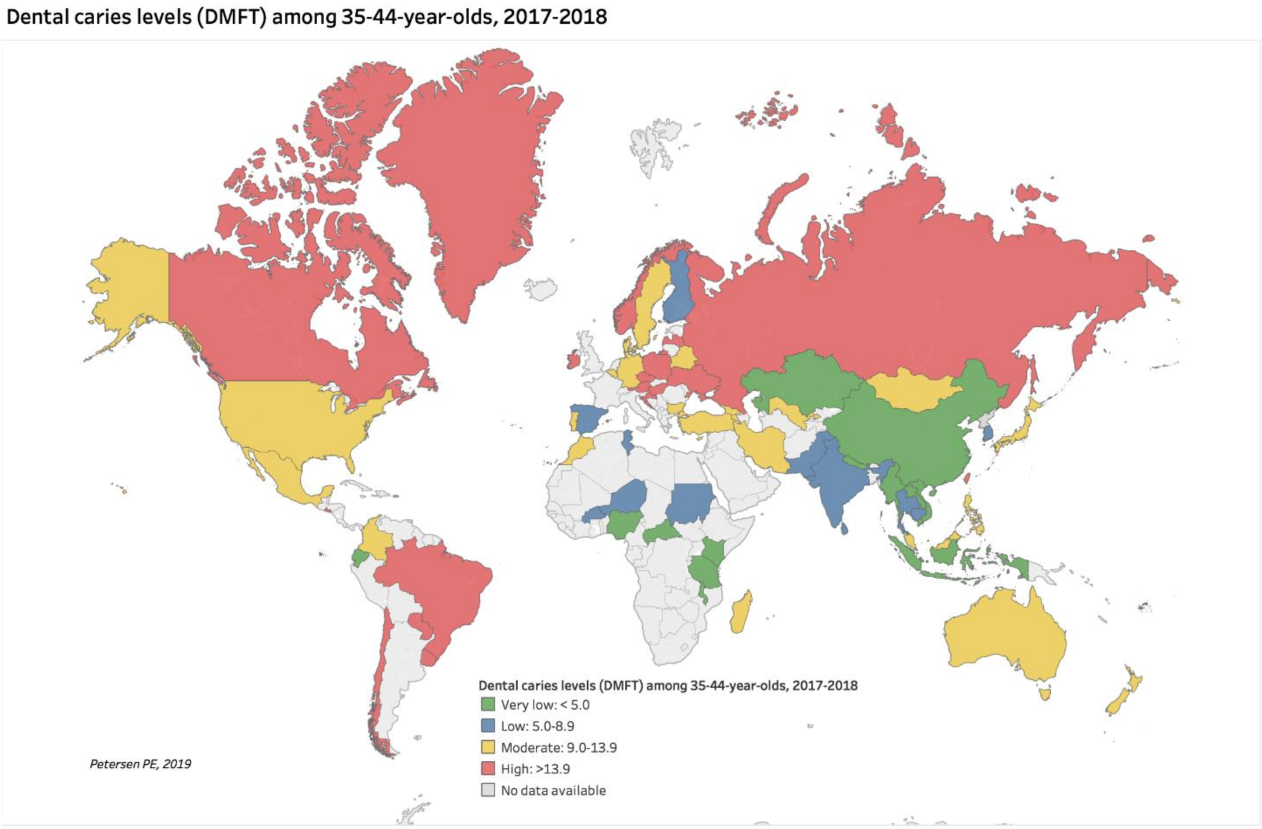

Fig. S4 Mean number of teeth (DMFT) affected by dental caries in adults aged 35-44 in countries across the world.

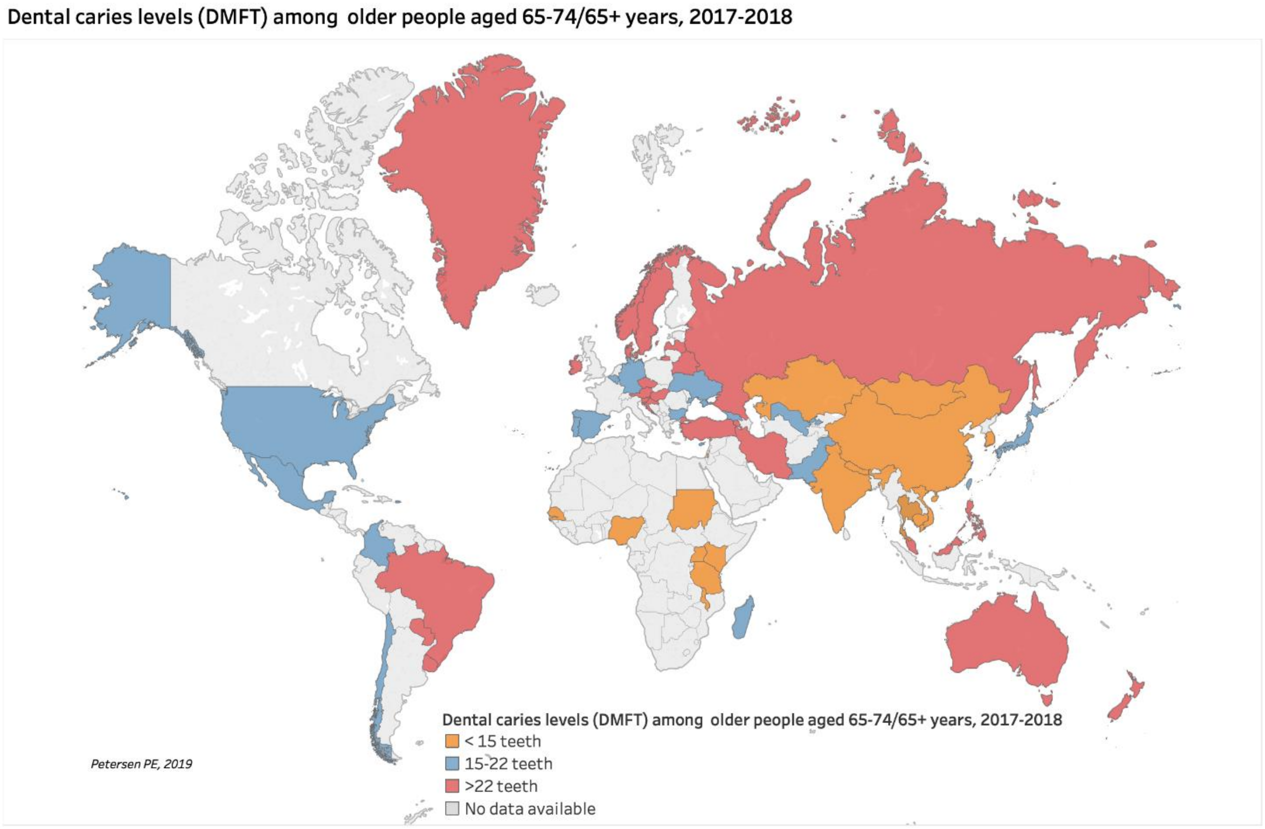

Fig. S5 Mean number of teeth affected by dental caries (DMFT) in older people aged 65-74/65+ years in countries across the world.

Pct. of 65-74/65+ year-olds with 20+ natural teeth, 2017-2018

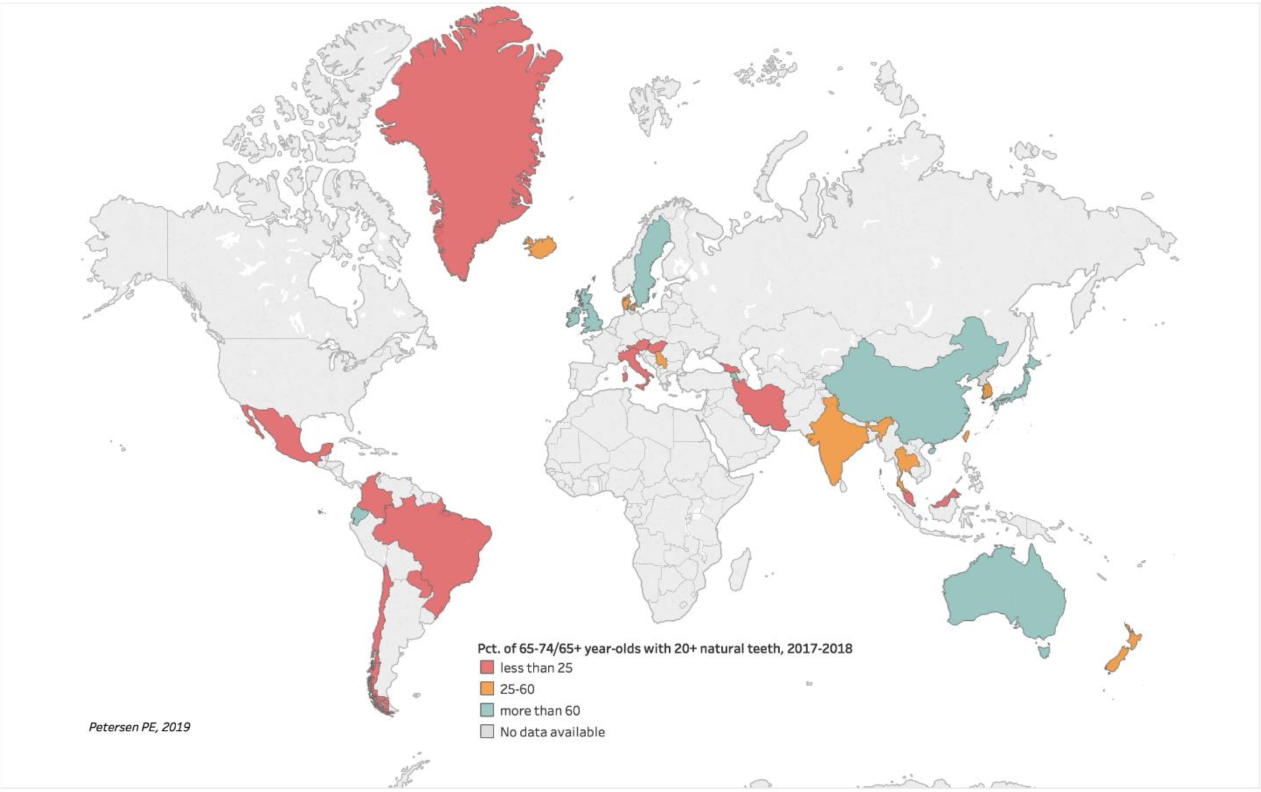

Fig. S6 Percentage of people aged 65-74/65+ years having at least 20 natural teeth (functional dentition) in countries across the world.

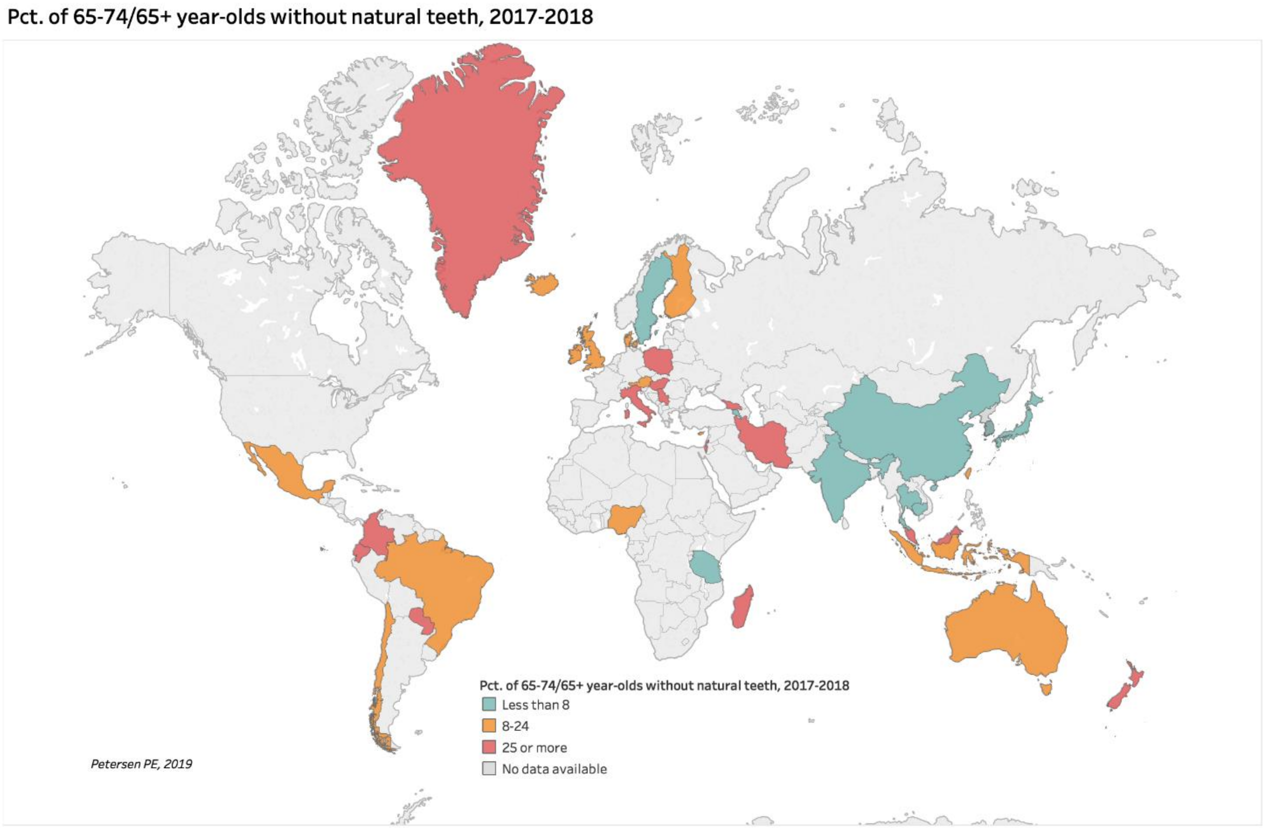

Fig. S7 Percentage of people aged 65-74/65+ years without natural teeth in countries across the world.
