# Supplementary material for: Global application of oral disease prevention and health promotion as measured 10 years after the 2007 World Health Assembly statement on oral health
Source: Community Dent Oral Epidemiol. 2020 May 8;48(4):338–48. doi: 10.1111/cdoe.12538 (PMC7496398; doi:10.1111/cdoe.12538)
Supplement: Supplementary file 2 — Table S1‐S16 [file CDOE-48-338-s002.pdf]

Global application of

# Oral Disease Prevention and Health Promotion

as measured ten years after the 2007 WHO World Health Assembly statement on oral health

Online supplementary information

Tables S1-S16

**Correspondence:**
Professor Poul Erik Petersen, DDS, Dr.Odont.Sci. BA, MSc (Sociology)
WHO Collaborating Centre for Community Oral Health Programmes and Research
University of Copenhagen
DK-1014 Copenhagen K, Denmark
poep@sund.ku.dk

**Table S1.**

Percentage of annual GNP spent on health/oral health (2015), mean number of registered dentists, percentage of dentists working in public and private sectors, population per dentist, and number of auxiliary personnel per dentist in low, middle and high-income countries.

| | Low-income (n=21) | Middle-income (n=39) | High-income (n=41) | Total (n=101) |
|---|---|---|---|---|
| Percentage of GNP spent on health | 6.23 | 6.84 | 8.79* | 7.59 |
| Percentage of GNP spent on oral health | 1.03 | 1.42 | 1.60 | 1.29 |
| Mean no. of registered dentists | 682 | 24209 | 21968 | 18213 |
| Pct. of dentists working in: | | | | |
| Public health service | 51.6 | 29.6 | 22.5 | 31.7 |
| Private dental practice | 33.7 | 59.7 | 69.1 | 57.4 |
| University dental schools | 8.9 | 6.3 | 4.7 | 6.3 |
| Related occupations | 5.8 | 2.9 | 2.1 | 3.5 |
| Population per dentist | 152721 | 13810 | 1708 | 90936 |
| Auxiliary personnel | | | | |
| No. of chairside assistants/ dental nurses per dentist | 0.02 | 0.3 | 1.5 | 0.8 |
| No. of oral hygienists per dentist | 0.2 | 0.1 | 0.8 | 0.5 |
| No. of therapists *(dental nurses with additional training in simple clinical work)* per dentist | 0.2 | 0.1 | 0.3 | 0.2 |

**Table S2.**

Percentage of countries where population groups receive care in oral health from government, financial support from government, or no support by level of national income.

| | Low-income (n=21) | Middle-income (n=39) | High-income (n=41) | Total (n=101) |
|---|---|---|---|---|
| Preschool children | | | | |
| Government care* | 9.5 | 15.4 | 31.7 | 20.8 |
| Financial support* | 4.8 | 25.6 | 17.1 | 17.8 |
| No support* | 85.7 | 59.0 | 51.2 | 61.4 |
| School children | | | | |
| Government care* | 9.5 | 15.4 | 34.1 | 21.8 |
| Financial support | 9.5 | 25.6 | 14.6 | 17.8 |
| No support* | 81.0 | 59.0 | 51.2 | 60.4 |
| Adolescents | | | | |
| Government care* | 4.8 | 10.3 | 34.1 | 18.8 |
| Financial support | 14.3 | 10.3 | 14.6 | 12.9 |
| No support* | 81.0 | 79.5 | 51.2 | 68.3 |
| Adults* | | | | |
| Government care | 4.8 | 7.7 | 19.5 | 11.9 |
| Financial support | 9.5 | 20.5 | 24.4 | 19.8 |
| No support | 85 .7 | 71.8 | 56.1 | 68.3 |
| Older people | | | | |
| Government care | 9.5 | 7.7 | 19.5 | 12.9 |
| Financial support | 9.5 | 23.1 | 19.5 | 18.8 |
| No support* | 81.0 | 69.2 | 61.0 | 68.3 |

*P<0.05

**Table S3.**

Percentage of countries where population groups receive financial support in oral health care from private health insurance at various points by level of national income.

| | Low-income (n=21) | Middle-income (n=39) | High-income (n=41) | Total (n=101) |
|---|---|---|---|---|
| Preschool children | | | | |
| 1-20% | 23.8 | 20.5 | 17.1 | 19.8 |
| 20-49% | 4.8 | - | 7.3 | 4.0 |
| 50-79% | 4.8 | 2.6 | 2.4 | 3.0 |
| 80%+ | 4.8 | - | - | 1.0 |
| No support | 61.9 | 76.9 | 73.2 | 72.3 |
| School children | | | | |
| 1-20% | 14.3 | 25.6 | 19.5 | 20.8 |
| 20-49% | 9.6 | - | 4.9 | 4.0 |
| 50-79% | 4.8 | 2.6 | 2.4 | 3.0 |
| 80%+ | 9.6 | - | - | 2.0 |
| No support | 61.9 | 71.8 | 73.2 | 70.3 |
| Adolescents | | | | |
| 1-20% | 14.3 | 23.1 | 17.1 | 18.8 |
| 20-49% | 9.5 | - | 7.3 | 5.0 |
| 50-79% | 4.8 | 2.6 | 2.4 | 3.0 |
| 80%+ | 9.6 | - | - | 2.0 |
| No support | 61.9 | 74.4 | 73.2 | 71.3 |
| Adults | | | | |
| 1-20% | 14.3 | 25.6 | 19.5 | 20.8 |
| 20-49% | 9.5 | 2.6 | 12.2 | 7.9 |
| 50-79% | 4.8 | 2.6 | 2.4 | 3.0 |
| 80%+ | 4.8 | - | - | 2.0 |
| No support | 61.9 | 69.2 | 65.9 | 66.3 |
| Older people | | | | |
| 1-20% | 23.8 | 25.6 | 22.0 | 23.8 |
| 20-49% | - | - | 9.8 | 4.0 |
| 50-79% | 4.8 | 2.6 | 2.4 | 3.0 |
| 80%+ | 9.6 | - | - | 2.0 |
| No support | 61.9 | 71.8 | 65.9 | 67.3 |

## Table S4.

Percentage of countries where population groups carry out direct patient payment for oral health care (full or partial) by level of national income.

| | Low-income (n=21) | Middle-income (n=39) | High-income (n=41) | Total (n=101) |
|---|---|---|---|---|
| Preschool children | 42.9 | 38.5 | 43.5 | 41.6 |
| School children | 42.9 | 41.0 | 41.5 | 41.6 |
| Adolescents | 42.9 | 46.2 | 43.9 | 44.6 |
| Adults | 42.9 | 46.2 | 56.1 | 49.5 |
| Older people | 42.9 | 43.6 | 51.2 | 46.5 |

**Table S5.**

Percentage of countries reporting that key populations are covered by primary health care and emergency care by level of national income.

| | Low-income (n=21) | Middle-income (n=39) | High-income (n=41) | Total (n=101) |
|---|---|---|---|---|
| Primary health care | | | | |
| Pre-school children* | 47.0 | 61.3 | 72.2 | 64.9 |
| Schoolchildren* | 52.4 | 71.8 | 75.4 | 65.1 |
| Adolescents* | 57.1 | 47.8 | 73.0 | 63.1 |
| Adults* | 34.8 | 44.6 | 58.6 | 47.5 |
| Older people** | 38.0 | 53.7 | 68.1 | 53.5 |
| Emergency care | | | | |
| Pre-school children*** | 36.7 | 62.3 | 92.6 | 75.0 |
| Schoolchildren *** | 43.3 | 47.5 | 92.9 | 70.6 |
| Adolescents *** | 35.0 | 50.8 | 90.5 | 70.3 |
| Adults* | 35.0 | 49.9 | 82.7 | 64.1 |
| Older people*** | 35.8 | 39.3 | 86.5 | 61.3 |

*P<0.05 **P<0.01 P<0.001

**Table S6.**

Percentage of countries providing specific preventive programmes for key population groups of children and adolescents by level of national income.

| Activities | Low-income (n=21) | Middle-income (n=39) | High-income (n=41) | Total (n=101) |
|---|---|---|---|---|
| Dental examinations | | | | |
| Preschool children | 42.9 | 66.7 | 65.9 | 61.4 |
| Schoolchildren* | 47.6 | 71.8 | 80.5 | 70.3 |
| Adolescents*** | 33.3 | 51.3 | 75.6 | 57.4 |
| Topical application of fluoride | | | | |
| Preschool children** | 28.6 | 59.0 | 68.3 | 56.4 |
| Schoolchildren*** | 33.3 | 82.1 | 75.6 | 69.3 |
| Adolescents ** | 19.0 | 33.3 | 56.1 | 39.6 |
| Oral hygiene instruction | | | | |
| Preschool children | 66.7 | 71.4 | 73.2 | 70.3 |
| Schoolchildren | 71.4 | 78.0 | 84.6 | 79.2 |
| Adolescents | 59.0 | 66.7 | 73.2 | 66.3 |
| Health education | | | | |
| Preschool children* | 61.9 | 64.1 | 80.5 | 70.3 |
| Schoolchildren* | 66.7 | 79.5 | 82.9 | 78.2 |
| Adolescents | 71.4 | 64.1 | 75.6 | 70.3 |

*P<0.05 **P<0.01 ***P<0.001

**Table S7.**

Percentage of countries providing selected preventive services for adults and older people in relation to level of national income.

| Activities | **Low-income (n=21)** | **Middle-income (n=39)** | **High-income (n=41)** | **Total (n=101)** |
|---|---|---|---|---|
| Dental examinations for adults* | 28.6 | 35.9 | 53.7 | 41.6 |
| Dental examinations for older people* | 23.8 | 30.8 | 58.5 | 40.6 |
| Topical application of fluoride for adults* | 14.3 | 12.8 | 29.3 | 19.8 |
| Topical application of fluoride for older people** | 14.3 | 10.3 | 39.0 | 22.8 |
| Oral hygiene instruction for adults | 41.0 | 53.7 | 61.9 | 50.5 |
| Oral hygiene instruction for older people | 46.2 | 61.9 | 61.0 | 55.4 |
| Health education for adults | 46.3 | 46.2 | 66.7 | 50.5 |
| Health education for older people | 43.6 | 56.1 | 66.7 | 53.5 |

*P<0.05 **P<0.01

## Table S8.

Percentage of countries having established school oral health programmes and percentage of countries with schools providing specific preventive services. Activities reported by level of national income.

| Activities | Low-income (n=21) | Middle-income (n=39) | High-income (n=41) | Total (n=101) |
|---|---|---|---|---|
| School oral health programme exists* | 57.1 | 76.9 | 76.3 | 72.4 |
| Fluoride mouth rinsing | | | | |
| Children <5 years | 16.7 | 10.0 | 13.8 | 12.7 |
| Children 5-7 years | 25.0 | 36.7 | 31.1 | 32.4 |
| Children aged 12 years | 25.0 | 36.7 | 31.0 | 32.4 |
| Adolescents aged 15 years | - | 10.0 | 13.8 | 9.9 |
| Pit and fissure sealing | | | | |
| Children <5 years | 25.0 | 13.3 | 13.8 | 15.5 |
| Children 5-7 years | 50.0 | 70.0 | 79.3 | 70.4 |
| Children aged 12 | 50.0 | 56.6 | 65.5 | 59.2 |
| Adolescents aged 15 * | - | 6.7 | 24.1 | 12.6 |
| Restorative care/treatment services | | | | |
| Children <5 years | 33.3 | 40.0 | 31.0 | 35.2 |
| Children 5-7 years * | 33.3 | 66.7 | 65.5 | 60.6 |
| Children aged 12 * | 41.7 | 76.7 | 62.1 | 64.8 |
| Adolescents aged 15 | 25.0 | 46.7 | 44.8 | 42.3 |

*P<0.05

**Table S9.**

Percentage of countries offering oral health education to children and adolescents as part of school oral health programmes. Activities reported by level of national income.

| Activities | Low-income (n=21) | Middle-income (n=39) | High-income (n=41) | Total (n=101) |
|---|---|---|---|---|
| Offer education in proper nutrition, diet and consumption of sugars | | | | |
| Children <5 years | 45.0 | 48.7 | 53.7 | 50.0 |
| Children 5-7 years | 60.0 | 64.1 | 63.4 | 63.0 |
| Children aged 12 | 60.0 | 66.7 | 58.5 | 62.0 |
| Adolescents aged 15 | 50.0 | 38.5 | 48.8 | 45.0 |
| Offer education in tobacco prevention | | | | |
| Children aged 12* | 35.0 | 43.6 | 53.7 | 46.0 |
| Adolescents aged 15* | 30.0 | 51.3 | 58.5 | 50.0 |
| Offer education in prevention of alcohol | | | | |
| Children aged 12 | 35.0 | 35.9 | 31.7 | 34.0 |
| Adolescents aged 15 | 35.0 | 41.0 | 39.0 | 39.0 |

*P<0.05

**Table S10.**

Percentage of countries with certain community approaches to oral health where activities are organized very often or often.

| Community approaches | **Low-income (n=21)** | **Middle-income (n=39)** | **High-income (n=41)** | **Total (n=101)** |
|---|---|---|---|---|
| Mass communication | 57.2 | 30.8 | 44.7 | 41.9 |
| Community campaigns/events | 52.4 | 48.7 | 55.3 | 52.1 |
| Maternity and child health facilities* | 28.6 | 43.6 | 55.2 | 44.9 |
| Work with families | 23.8 | 25.7 | 32.1 | 31.7 |
| Working place activities | 19.1 | 15.4 | 13.2 | 15.3 |
| Programmes for older people** | 4.8 | 10.2 | 34.2 | 18.3 |

*P<0.05 **P<0.01

**Table S11.**

Percentage of countries with national health programmes for prevention of NCDs considering oral health.

| | Low-income (n=21) | Middle-income (n=39) | High-income (n=41) | Total (n=101) |
|---|---|---|---|---|
| Diabetes | 52.4 | 64.1 | 50.0 | 56.1 |
| Cardiovascular diseases | 47.6 | 46.2 | 34.2 | 41.8 |
| Tobacco* | 42.9 | 69.2 | 68.4 | 63.3 |
| Alcohol | 33.3 | 35.9 | 36.8 | 35.7 |
| Nutrition | 66.7 | 59.0 | 55.3 | 59.2 |
| Diet and sugars* | 52.4 | 46.2 | 68.4 | 56.1 |
| National policy or recommendations given to reduce the intake of sugars** | 23.8 | 51.3 | 76.3 | 55.1 |

*P<0.05 **P<0.01

## Table S12.

Mean percentages of national populations benefitting from fluoride programmes, percentage of countries with political interest in fluoridation programmes, percentage of countries with necessary conditions of providing fluoridation schemes, and percentage of countries planning the introduction of population methods of fluoridation. Activities presented by level of national income.

| | Low-income (n=21) | Middle-income (n=39) | High-income (n=41) | Total (n=101) |
|---|---|---|---|---|
| Mean percentages of population who benefits from fluoride | | | | |
| Drinking water | - | 7.6 | 20.9 | 11.2 |
| Fluoridated salt | 2.5 | 9.8 | 7.6 | 7.4 |
| Fluoridated milk | - | 2.7 | 0.01 | 1.1 |
| Toothpaste containing fluoride *** | 30.8 | 52.1 | 81.5 | 59.1 |
| Political interest good/very good | | | | |
| Water fluoridation | 28.6 | 17.9 | 35.1 | 26.8 |
| Salt fluoridation | 25.0 | 12.9 | 13.6 | 15.6 |
| Milk fluoridation | 5.0 | 17.9 | 8.1 | 11.5 |
| Conditions for population methods of fluoridation | | | | |
| Burden of dental caries | 28.6 | 59.0 | 44.7 | 46.9 |
| Technical requirements and facilities** | 9.5 | 33.3 | 34.2 | 28.6 |
| Necessary expertise** | 9.5 | 46.2 | 39.5 | 35.7 |
| Public acceptance | 23.8 | 28.2 | 34.2 | 29.6 |
| Political interest | 23.8 | 25.6 | 28.9 | 26.5 |
| Plans for population methods of fluoridation | | | | |
| Water | 19.0 | 15.4 | 15.8 | 16.3 |
| Salt | 20.0 | 12.8 | 7.9 | 12.4 |
| Milk | - | 12.8 | 2.6 | 6.2 |

**P<0.01 ***P<0.001

## Table S13.

Percentage of countries with specific fluoride recommendation of toothpaste according to level of national income.

| | Low-income (n=21) | Middle-income (n=39) | High-income (n=41) | Total (n=101) |
|---|---|---|---|---|
| Toothpaste containing fluoride | | | | |
| 400-600 ppm F | | | | |
| Pre-school children | 40.0 | 38.5 | 44.7 | 41.2 |
| Schoolchildren* | 15.0 | 10.3 | 2.6 | 8.2 |
| 1000-1500 ppm F | | | | |
| Pre-school children** | 5.0 | 25.6 | 47.4 | 29.9 |
| Schoolchildren** | 35.0 | 61.5 | 86.8 | 66.0 |
| Adults** | 35.0 | 46.2 | 76.3 | 55.7 |
| >1500 ppm F | | | | |
| Adults | 20.0 | 23.1 | 15.8 | 19.5 |
| No specific recommendation** | 45.0 | 30.8 | 7.9 | 24.8 |

*P<0.05 **P<0.01

## Table S14.

Percentage of countries having national targets for oral health and established information systems for surveillance of oral health by level of national income.

| Targets and surveillance | Low-income (n=21) | Middle-income (n=39) | High-income (n=41) | Total (n=101) |
|---|---|---|---|---|
| Targets formulated for key population groups | | | | |
| Children aged 5-6 | 40.0 | 51.3 | 44.7 | 46.4 |
| Children aged 12 | 30.0 | 56.4 | 50.4 | 48.5 |
| Adolescents aged 18 | 25.0 | 23.1 | 15.8 | 20.6 |
| Adults aged 35-44 | 30.0 | 23.1 | 23.7 | 24.7 |
| Older people aged 65-74/65+ | 20.5 | 23.7 | 21.6 | 20.0 |
| National information systems for surveillance of oral health | | | | |
| Children (5-6 and 12 years) | 42.9 | 61.5 | 63.2 | 58.2 |
| Youth (15-19 years) | 42.9 | 51.3 | 52.6 | 50.0 |
| Adults (35-44 years) | 42.9 | 30.8 | 44.7 | 38.8 |
| Older people (65-74/65+ years) | 38.1 | 30.8 | 39.5 | 35.7 |

**Table S15.**

Epidemiological indicators of oral health in different WHO standard age groups by level of national income.

| Indicators of dental caries (DMFT) | **Low-income** | **Middle-income** | **High-income** | **Total** |
|---|---|---|---|---|
| Average percentage of caries free children aged 5 or 6 *** | 36.3 (n=13) | 28.9 (n=53) | 50.2 (n=43) | 38.2 (n=109) |
| Average DMFT in children aged 12 * | 1.3 (n=12) | 2.1 (n=64) | 1.6 (n=50) | 1.9 (n=126) |
| Average DMFT in adolescents aged 18 | 2.8 (n=10) | 4.1 (n=25) | 4.0 (n=22) | 3.8 (n=57) |
| Average DMFT in adults aged 35-44 years *** | 4.9 (n=10) | 9.6 (n=33) | 12.1 (n=28) | 10.1 (n=71) |
| Average DMFT in older people aged 65-74/65+ *** | 8.0 (n=5) | 17.1 (n=26) | 21.0 (n=26) | 18.1 (n=57) |

*P<0.05 ***P<0.001

## Table S16.

Epidemiologic indicators on dentate status and periodontal health status (CPI) of different WHO standard age groups by level of national income. Percentage of people with different dental health conditions.

| Indicators | **Low-income** | **Middle-income** | **High-income** | **Total** |
|---|---|---|---|---|
| Dentate status | | | | |
| 35-44 years without natural teeth | 3.5 (n=2) | 4.8 (n=19) | 0.8 (n=21) | 2.7 (n=42) |
| 35-44 year with 20+ teeth | 74.7 (n=3) | 78.6 (n=17) | 92.4 (n=16) | 84.4 (n=36) |
| 65-74 years without natural teeth | 15.6 (n=4) | 18.3 (n=21) | 17.2 (n=23) | 17.5 (n=48) |
| 65-74 years with 20+ teeth | 59.5 (n=1) | 39.7 (n=18) | 50.1 (n=19) | 45.4 (n=38) |
| Periodontal status | | | | |
| 15-19 years | | | | |
| Gingival bleeding (scores 1+2) | 43.9 (n=4) | 49.8 (n=18) | 47.1 (n=9) | 48.3 (n=31) |
| Shallow pockets (score 3) | 11.7 (n=3) | 4.5 (n=15) | 6.4 (n=10) | 5.9 (n=28) |
| Deep pockets (score 4) | 6.7 (n=3) | 1.7 (n=13) | 1.4 (n=9) | 2.2 (n=25) |
| 35-44 years | | | | |
| Gingival bleeding (scores 1+2) | 33.7 (n=3) | 49.4 (n=18) | 46.2 (n=14) | 46.8 (n=35) |
| Shallow pockets (score 3) | 18.6 (n=3) | 20.9 (n=17) | 29.2 (n=16) | 24.4 (n=36) |
| Deep pockets (score 4) | 9.2 (n=3) | 8.0 (n=17) | 10.3 (n=15) | 9.1 (n=35) |
| 65-74 years | | | | |
| Gingival bleeding (scores 1+2) | 45.7 (n=2) | 39.4 (n=18) | 33.4 (n=14) | 37.3 (n=34) |
| Shallow pockets (score 3) | 24.7 (n=2) | 27.8 (n=16) | 39.6 (n=17) | 33.3 (n=35) |
| Deep pockets (score 4) | 24.9 (n=2) | 13.8 (n=16) | 20.1 (n=16) | 17.4 (n=34) |
